# Peristomal Pseudoverrucous Lesions: A Rare Skin Complication of Colostomy

**DOI:** 10.7759/cureus.38068

**Published:** 2023-04-24

**Authors:** Işıl D Oğuz, Selahattin Vural, Esma Cinar

**Affiliations:** 1 Dermatology, Giresun University Faculty of Medicine, Giresun, TUR; 2 General Surgery, Giresun University Faculty of Medicine, Giresun, TUR; 3 Pathology, Giresun University Faculty of Medicine, Giresun, TUR

**Keywords:** ostomy care, pseudoverrucous lesions, pseudoepitheliomatous hyperplasia, peristomal skin, colostomy

## Abstract

A 56-year-old female patient with colostomy presented with skin-colored cobblestone and verrucous asymptomatic papules on her peristomal skin for three months; she was referred to dermatology. Histopathology revealed irregular acanthosis, tongue-like extension of rete ridges of mature squamous epithelium without atypical morphology, hyperkeratosis, and inflammation of the skin. The histopathologic appearance was evaluated as compatible with pseudoepitheliomatous hyperplasia. No signs of malignancy, fungus, or koilocytes were found. The lesions were diagnosed as pseudoepitheliomatous hyperplasia by clinical and histopathologic findings. In this case report, we review pseudoepitheliomatous hyperplasia associated with colostomy.

## Introduction

Ostomy is an artificial opening surgically created between the bowel and the abdominal wall. It allows the excretion of feces or urine by the abdominal wall through the opening. After the procedure, an ostomy bag is attached to the peristomal skin to prevent stool or urine from flowing out [1]. Although it is a common procedure, it may cause some complications and impair quality of life. The most frequently reported complications associated with stoma are skin complications [2]. Peristomal skin complications have been reported between 18% and 55% in the literature. These complications may be due to mechanical, infectious, allergic, and chemical causes [3]. Skin contact with the ostomy bag, fecal and urinary contents, local friction, moisture accumulation, and trauma predispose this area to inflammatory and infectious skin disorders. Peristomal skin conditions include erythema, skin erosions, ulcers, folliculitis, candidiasis, irritant and allergic contact dermatitis, suture granulomas, pyoderma gangrenosum, and pseudoverrucous lesions [4,5]. In this case report, we discuss a rare presentation of pseudoepitheliomatous hyperplasia associated with colostomy.

## Case presentation

**Figure 1:**
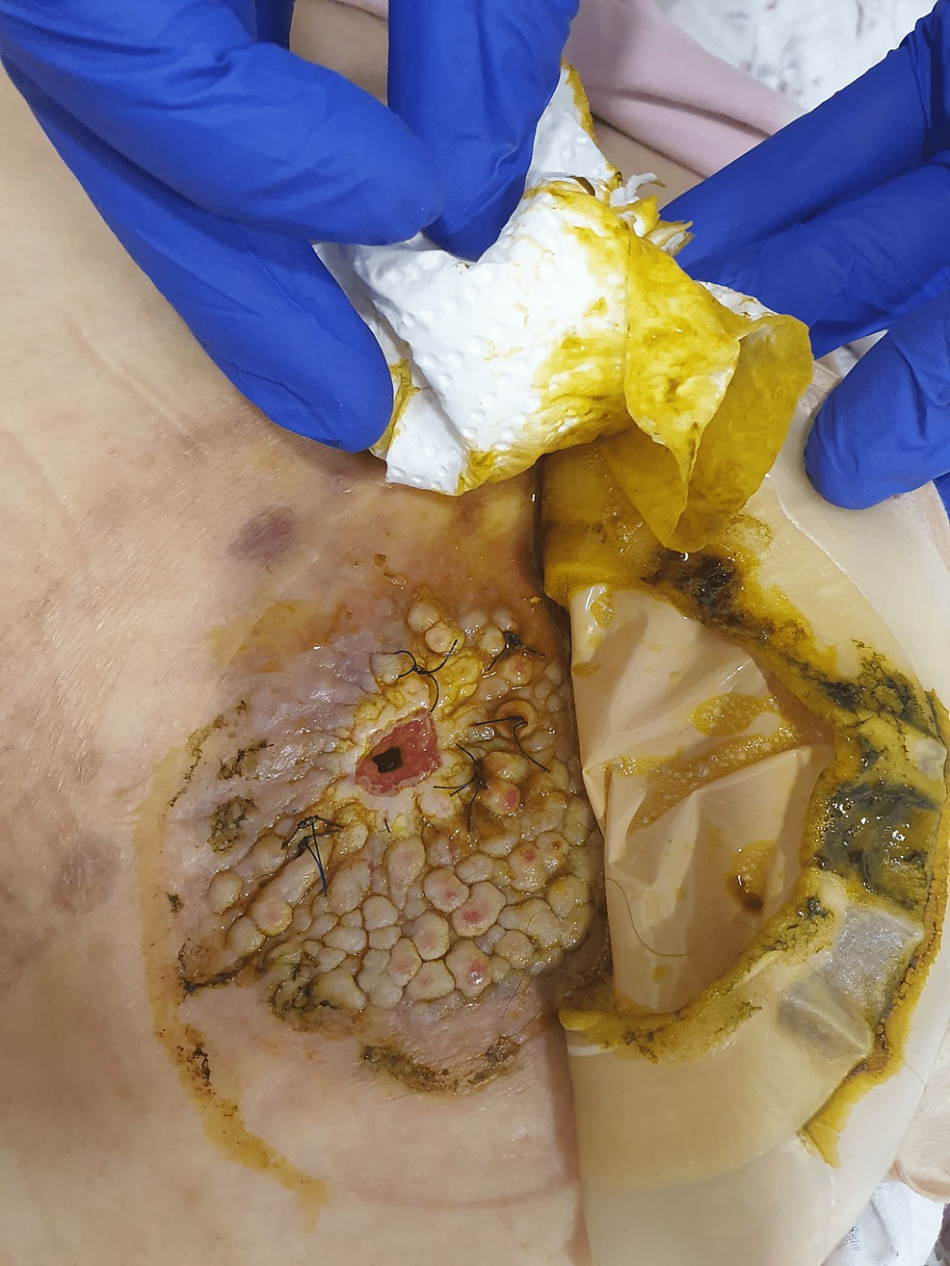
Multiple skin-colored cobblestone and verrucous papules on the peristomal skin

A 56-year-old female patient underwent a colostomy procedure six months after mesenteric artery ischemia. The patient reported to the emergency department three days earlier complaining of abdominal pain and inability to have a bowel movement. She was admitted to the surgical department with a diagnosis of ileus, which was resolved after the administration of an enema. On follow-up examination, multiple fungi-like papules were noted on the peristomal skin. Therefore, a dermatology consultation was requested. Her medical history revealed that she was taking anticoagulants, quetiapine, and sertraline due to chronic venous insufficiency, lymphedema, and an anxiety disorder, respectively. The patient had the described peristomal lesions for three months. Subjective symptoms such as itching and pain did not occur. Dermatologic examination revealed multiple skin-colored cobblestone and verrucous papules that did not extend beyond the boundaries of the colostomy bag and filled the entire peristomal skin in the colostomy bag (Figure 1).

**Figure 2:**
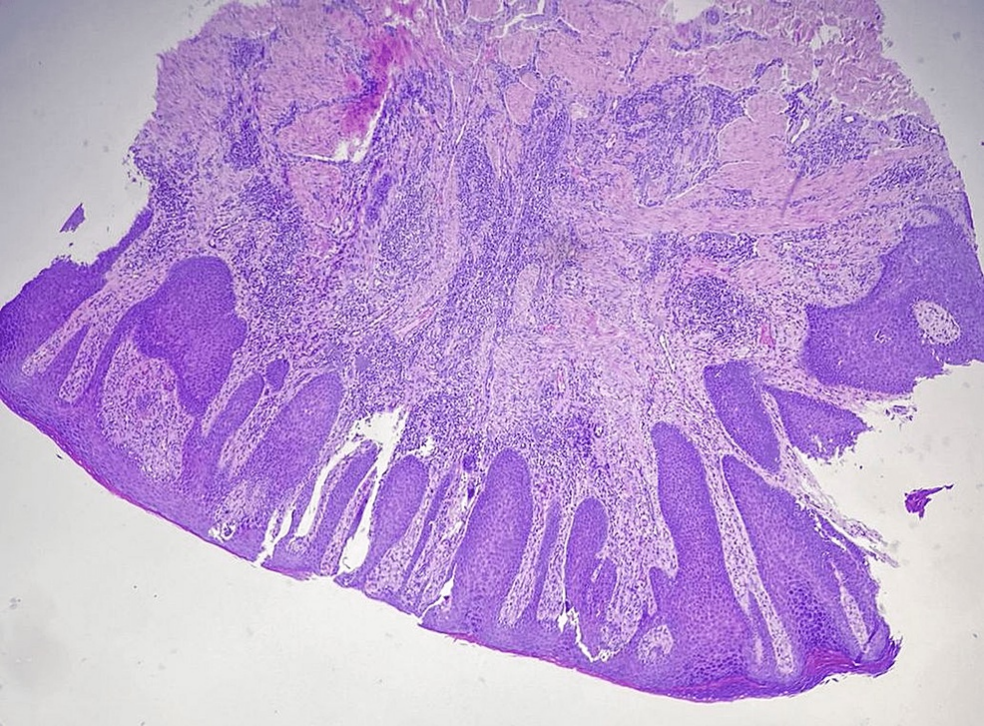
Irregular acanthosis, hyperkeratosis tongue-like extension of rete ridges of mature squamous epithelium, H&E x4

**Figure 3:**
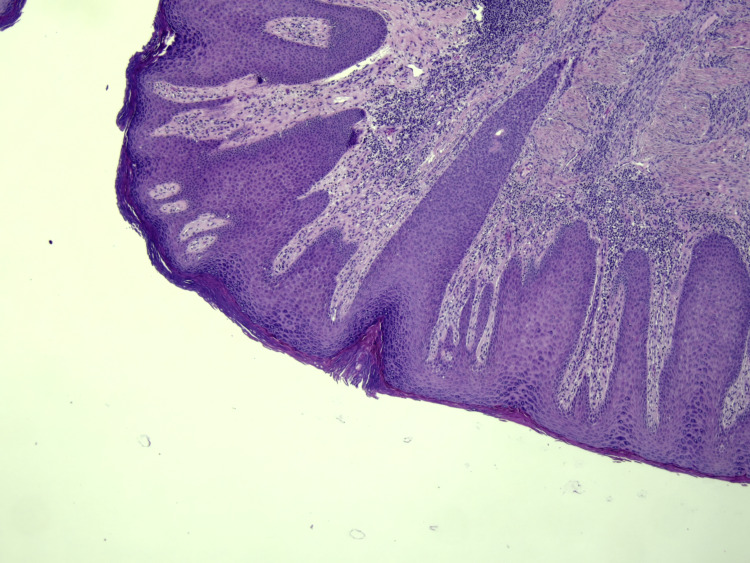
Irregular acanthosis, hyperkeratosis tongue-like extension of rete ridges of mature squamous epithelium, H&E x10

**Figure 4:**
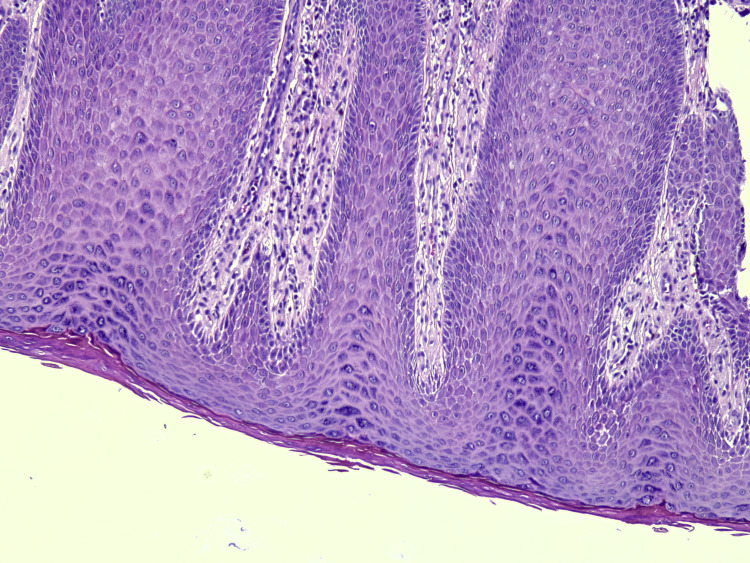
Pseudoepitheliomatous hyperplasia, H&E x20

**Figure 5:**
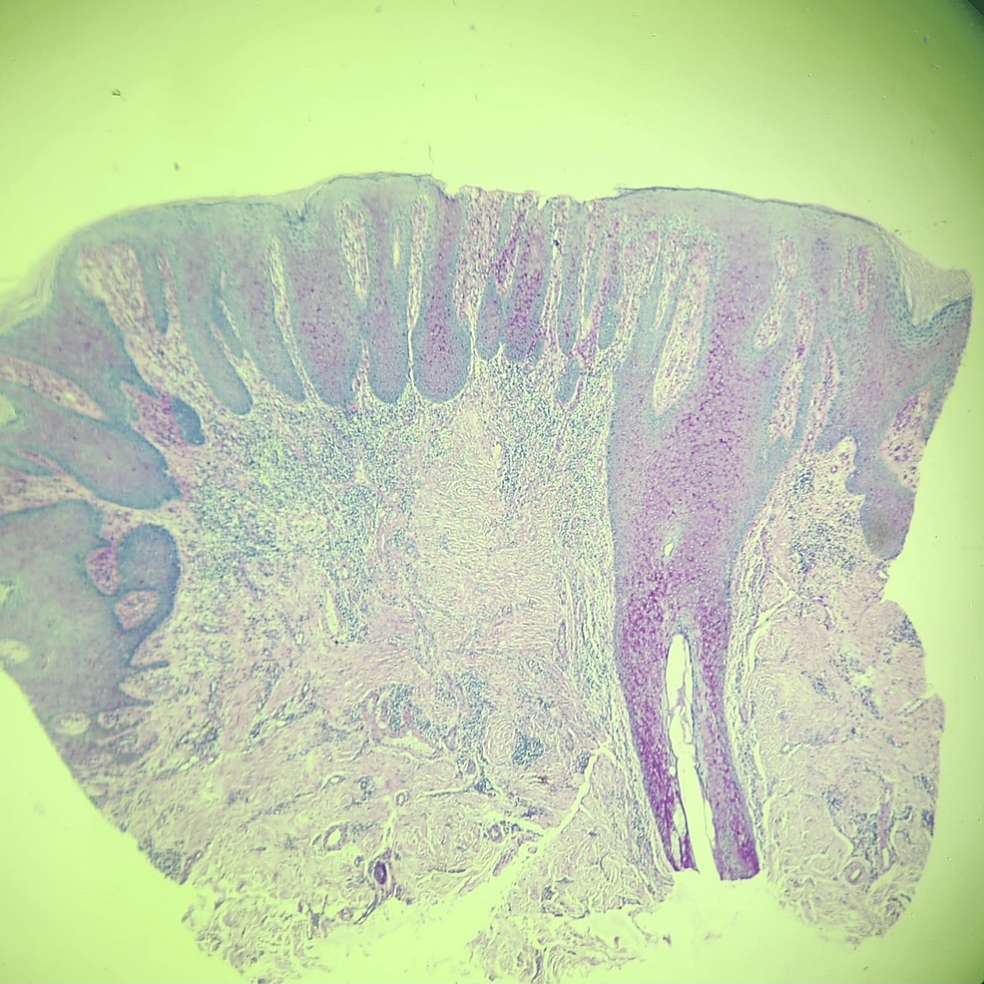
The basement membrane was intact; there were no fungal hyphae or spores in the epidermis and dermis, periodic acid-Schiff (PAS) x4

A punch biopsy was performed with the provisional diagnosis of cutaneous tumor, cutaneous metastases, condyloma, deep fungal infection, or pseudoepitheliomatous hyperplasia. Histopathology revealed irregular acanthosis, tongue-like extension of rete ridges of mature squamous epithelium without atypical morphology, hyperkeratosis, and inflammation of the skin. No signs of malignancy and fungal hyphae or spores were found. No koilocytes were noted, indicating human papillomavirus (HPV) effect. The histopathologic appearance was evaluated as compatible with pseudoepitheliomatous hyperplasia (Figures 2-5).

The colostomy bag was replaced with a smaller orifice. However, since the patient did not visit the hospital for follow-up and could not be reached, the current status of her lesions is unknown.

## Discussion

Peristomal pseudoverrucous lesions are usually detected around urostomies or ileal conduits that allow urinary excretion. Peristomal pseudoverrucous lesions may occur in approximately 20% of patients with stomas. Among stomas, peristomal pseudoverrucous lesions are reported in 22%-23% with urinary diversions [6,7]. Wide-opening ostomy bags cause urine leakage on the skin [6]. Under the influence of alkaline urine or infection, uric acid crystals accumulate in the peristomal skin and cause chronic inflammation in this region. As a result, wart-like appendages form on the peristomal skin [4,6]. Pseudoverrucous lesions may also develop around the ileostomy because of its high fluid volume [8].

Goldberg et al. [9] reported five children with perianal multiple flat-topped, smooth, red, round papules and nodules. Acanthosis or psoriasiform spongiotic dermatitis was observed on histopathologic examination. The common finding in all children was encopresis or urinary incontinence. Healing of the lesions when fecal or urinary contact was prevented indicated that the lesions developed because of chronic irritation by urine and feces.

Only three cases of colostomy-associated pseudoverrucous lesions have been reported in the literature [10-12]. Of the three patients, only one had pseudoepitheliomatous hyperplasia on histopathologic examination [12]. The biopsy traces of the other two patients showed histological features of eccrine syringofibroadenoma [10,11].

The first case of colostomy-associated pseudoverrucous lesions was reported in 1981 by Hjorth et al. They presented the case of a 51-year-old man with sigmoid colostomy after rectal adenocarcinoma surgery [12]. After radiotherapy, the patient received topical corticosteroids on the peristomal skin intermittently for 14 months because of oozing dermatitis. However, 2-6 mm red, firm, sparse nodules developed around the colostomy. Histopathologic examination revealed irregular acanthotic, hyperkeratotic, and parakeratotic epidermis and thickened dermis with perivascular inflammatory cells. Also in the stratum spinosum keratinocytes, periodic acid-Schiff (PAS)-positive granules were detected. This condition resembled granuloma gluteale infantum with clinic appearance and histopathologic findings. It has been suggested that these nodular lesions may be a chronic irritation of peristomal skin by intestinal contents. There are some differences between this case and the case we presented. In our case, we observed wart-like confluent lesions that filled the entire peristomal skin in the colostomy bag. In contrast, in the case reported by Hjorth et al., the lesions were sparse and resembled granuloma gluteale infantum. Despite the comparable histopathologic findings, we were unable to detect fungi by PAS staining, as observed by Hjorth et al. [12].

The other two cases were reported in 2003 [10] and 2005 [11]. The reason for colostomy placement in these patients was inflammatory bowel disease and rectal adenocarcinoma, respectively. Both patients had only one firm gray nodule on the peristomal skin. Histopathology of the lesions in both patients revealed an eccrine syringofibroadenoma.

Our patient is unique in that she differs from other reported cases in terms of the clinical and histopathologic features, as well as the location of the lesions.

Pseudoepitheliomatous hyperplasia (also called chronic papillomatous dermatitis or pseudoverrucous lesions) is a benign skin disease clinically manifested as skin-colored or pale pink verrucous papulonodules, while histopathology shows hypergranulotic and hyperkeratotic epidermis extending into the dermis in the form of finger-like protrusions. This condition may be associated with infections, neoplasms, chronic inflammation, and irritation [13]. Epidermal growth factor (EGF), transforming growth factor-α, and EGF receptors are thought to play a role in the pathophysiology of PEH. Increased expression of these mediators by the inflammatory infiltrate leads to the proliferation of the epidermis into the dermis [14].

In the differential diagnosis of pseudoverrucous lesions, diseases such as squamous cell carcinoma, cutaneous metastases, and other neoplastic diseases, condyloma, bacterial infections, and Candida should be considered [12,15]. Therefore, we recommend histopathologic examination to rule out malignancy in such lesions.

It is recommended that the opening of the ostomy bag be of adequate size and that the stoma protrude 1-3 mm to avoid chronic irritation and associated pseudoverrucous lesions [7]. Urinary acidification (for urostomies), application of silver nitrate, and surgical excision are recommended for treatment [6].

## Conclusions

Peristomal pseudoverrucous lesions are usually observed around the urostomy; however, it is seen around the colostomy in extremely rare conditions. Our patient is the first reported case in the literature that had peristomal wart-like clobstone lesions filling the entire peristomal skin. Multiple papular lesions may be confused with malignancies, condyloma, and fungal infections because they rarely occur around the colostomy. Therefore, pseudoverrucous lesions should also be kept in mind as a preliminary diagnosis in patients presenting with such lesions.

## References

[1] Morss-Walton PC, Yi JZ, Gunning ME, McGee JS (2021). Ostomy 101 for dermatologists: managing peristomal skin diseases. Dermatol Ther.

[2] Murken DR, Bleier JI (2019). Ostomy-related complications. Clin Colon Rectal Surg.

[3] Kwiatt M, Kawata M (2013). Avoidance and management of stomal complications. Clin Colon Rectal Surg.

[4] Woo KY, Sibbald RG, Ayello EA, Coutts PM, Garde DE (2009). Peristomal skin complications and management. Adv Skin Wound Care.

[5] D'Ambrosio F, Pappalardo C, Scardigno A, Maida A, Ricciardi R, Calabrò GE (2022). Peristomal skin complications in ileostomy and colostomy patients: what we need to know from a public health perspective. Int J Environ Res Public Health.

[6] Almutairi D, LeBlanc K, Alavi A (2018). Peristomal skin complications: what dermatologists need to know. Int J Dermatol.

[7] Brogna L (2021). Prevention and management of pseudoverrucous lesions: a review and case scenarios. Adv Skin Wound Care.

[8] Hocevar BJ (2010). WOC nurse consult: moist, painful peristomal skin. J Wound Ostomy Continence Nurs.

[9] Goldberg NS, Esterly NB, Rothman KF (1992). Perianal pseudoverrucous papules and nodules in children. Arch Dermatol.

[10] Clarke LE, Ioffreda M, Abt AB (2003). Eccrine syringofibroadenoma arising in peristomal skin: a report of two cases. Int J Surg Pathol.

[11] Kazakov DV, Mikyskova I, Mukensnabl P, Brouckova M, Treska V, Hes O, Michal M (2005). Reactive syringofibroadenomatous hyperplasia in peristomal skin with formation of hybrid epidermal-colonic mucosa glandular structures, intraepidermal areas of sebaceous differentiation, induction of hair follicles, and features of human papillomavirus infection: a diagnostic pitfall. Am J Dermatopathol.

[12] Hjorth N, Sjølin KE (1981). Multiple inflammatory acanthomas around a colostomy. J Cutan Pathol.

[13] Zayour M, Lazova R (2011). Pseudoepitheliomatous hyperplasia: a review. Am J Dermatopathol.

[14] El-Khoury J, Kibbi AG, Abbas O (2012). Mucocutaneous pseudoepitheliomatous hyperplasia: a review. Am J Dermatopathol.

[15] Alslaim F, Al Farajat F, Alslaim HS, Drevets P, Jenkins B (2021). Etiology and management of peristomal pseudoepitheliomatous hyperplasia. Cureus.

